# Efficacy of bilateral quadratus lumborum block with liposomal bupivacaine for post-cesarean analgesia: a protocol for an open-label randomized controlled trial

**DOI:** 10.3389/fmed.2025.1654019

**Published:** 2025-09-23

**Authors:** Wenru Ma, Yinhuan Liu, Yan Li, Kongjuan Zhu, Youjia Yu, Yanting Wang

**Affiliations:** ^1^Department of Anesthesiology, The Affiliated Hospital of Qingdao University, Qingdao, China; ^2^Department of Anesthesiology and Pain Medicine, Suzhou Xiangcheng People’s Hospital, Suzhou, China

**Keywords:** analgesia, caesarean section, liposomal bupivacaine, pain, quadratus lumborum

## Abstract

**Background:**

Effective postoperative pain management is essential for optimizing maternal comfort and recovery following cesarean delivery. However, achieving adequate analgesia remains a challenge due to suboptimal pain control and medication-related adverse effects. This study aims to evaluate the efficacy of bilateral quadratus lumborum block using liposomal bupivacaine for post-cesarean analgesia.

**Methods:**

This single-center, open-label, randomized controlled trial will enroll 201 adult parturients undergoing lower segment transverse cesarean section. Participants will be randomized in a 1:1:1 ratio into three groups (*n* = 67 each): Group LB will receive bilateral quadratus lumborum block with liposomal bupivacaine under ultrasound guidance. Group R-P will receive bilateral quadratus lumborum block with ropivacaine in combination with patient-controlled intravenous analgesia. Group P will receive standard patient-controlled intravenous analgesia without regional nerve block. The primary outcome is postoperative pain intensity, assessed using the Visual Analogue Scale during standardized movement at 12, 24, 48, and 72 h postoperatively. A generalized estimating equation model will be used to assess the overall effect of treatment group on pain scores over time. Secondary outcomes include patient-reported Quality of Recovery-15 scores at 24 and 48 h postoperatively; Visual Analogue Scale for pain at rest, measured at 12, 24, 48, and 72 h postoperatively; need for rescue analgesia; postoperative anxiety (assessed by Visual Analogue Scale at 72 h); subjective sleep quality; maximum walking duration; patient satisfaction with analgesia; incidence of nausea and vomiting; postoperative sufentanil consumption and length of hospital stay. Safety outcomes include allergic reactions, wound infection, lightheadedness, headache, circumoral numbness, tongue paresthesia, drowsiness, irritability, blood pressure fluctuations (hypotension, hypertension), respiratory depression (hypoventilation), and cardiac events (bradycardia, tachycardia, arrhythmia, cardiac arrest). Data will be analyzed using a modified intention-to-treat approach.

**Discussion:**

This study aims to provide high-quality evidence on the efficacy of bilateral single-injection quadratus lumborum block with liposomal bupivacaine, compared with standard intravenous analgesia alone and combined with quadratus lumborum block using ropivacaine.

**Ethics and dissemination:**

Ethics approval was obtained from the Ethics Committee of the Affiliated Hospital of Qingdao University (QYFYEC2024-297). All parturients will provide written informed consent. The results of this study will be published in a peer-reviewed journal.

**Clinical trial registration:**

Identifier ChiCTR2500095835.

## 1 Introduction

Cesarean section represents one of the most common surgical procedures worldwide (1, 2). It is common for parturients of reproductive age to experience significant anxiety regarding the prospect of postoperative pain following a cesarean section, which often takes precedence over other potential postoperative concerns (3). Effective postoperative analgesia is crucial for optimizing recovery, facilitating early maternal mobilization, reducing hospital length of stay, and promoting breastfeeding initiation (4, 5). Moreover, adequate analgesia not only strengthens maternal–infant bonding but also reduces the risk of postpartum depression, ultimately contributing to the overall well-being and psychological resilience of mothers during the critical postnatal period (6, 7).

In medical practice, clinicians continuously refine postoperative analgesic protocols to enhance both effectiveness and practicality. Patient-controlled analgesia (PCA) pumps have historically been the primary modality for postoperative pain management and remain widely used today, delivering pharmacologic agents via indwelling catheters to epidural, intravenous, and fascial plane compartments (8–10). Although epidural analgesia remains a clinically well-established modality, its implementation frequently elicits patient anxiety and procedural discomfort. Serious complications such as neurological injury and epidural hematoma, along with common adverse events including hypotension, urinary retention, and infection (11). Notably, evidence associates epidural techniques with heightened risks of cardiovascular complications, particularly myocardial infarction and cardiac arrest (12). Patient-controlled intravenous analgesia (PCIA), while demonstrating efficacy in postoperative pain management, presents a distinct adverse effect profile. Opioids administration correlates with nausea/vomiting rates exceeding 50% (13). In contrast, regional nerve blocks with local anesthetics demonstrates dual clinical benefits: a significant reduction in opioid consumption and a lower incidence of systemic adverse events compared to conventional analgesia approaches. However, a critical limitation persists: current local anesthetics fail to provide analgesia of sufficient duration, which fundamentally restricts the clinical applicability of single-injection regional nerve blocks for postoperative pain management, despite their proven intraoperative performance.

Liposomal bupivacaine, an innovative sustained-release local anesthetic with a favorable safety profile (14), utilizes multivesicular liposomal technology to encapsulate bupivacaine within phospholipid vesicles. This structural characteristic enables gradual drug release, achieving extended analgesic duration while maintaining therapeutic concentrations (15). Additionally, existing evidence indicates that bilateral quadratus lumborum (QL) blocks provide more effective postoperative pain relief than transversus abdominis plane (TAP) blocks—well-established regional anesthesia technique (16–18). Given the prolonged analgesic duration of liposomal bupivacaine and the superior efficacy of QL blocks, this study aims to evaluate the efficacy of bilateral quadratus lumborum block with liposomal bupivacaine for post-cesarean analgesia.

## 2 Methods

This protocol adheres to the Standard Protocol Items: Recommendations for Interventional Trials (SPIRIT) guidelines.

### 2.1 Study design and patients

**Figure 1:**
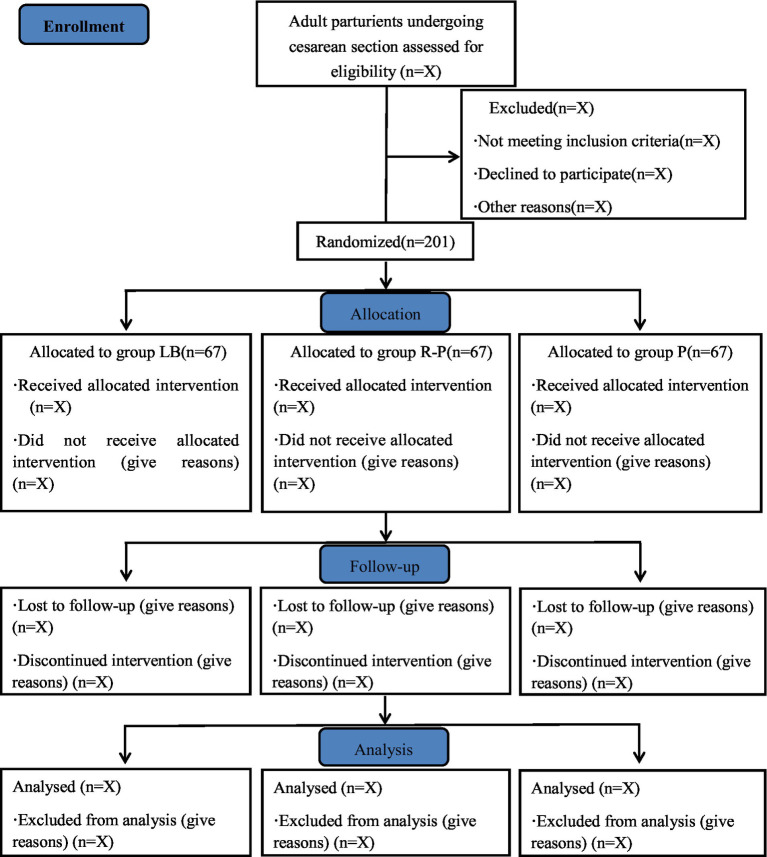
Study flow diagram. Grouping according to modes of Intervention. LB, liposomal bupivacaine; R, ropivacaine; P, patient-controlled intravenous analgesia pump.

This study is a single-center, prospective, randomized, open-label, parallel-group clinical trial. The trial will be conducted at the Affiliated Hospital of Qingdao University, enrolling a total of 201 patients. Recruitment is scheduled to take place from 20th January 2025 to 10th December 2025. The study flow diagram is shown in Figure 1.

### 2.2 Inclusion criteria

Patients who meet the following criteria will be included.

- Aged 18 and above;
- Singleton pregnancy;
- Scheduled for elective cesarean section;
- Individuals who provide consent to participate in this study and sign the informed consent document.

### 2.3 Exclusion criteria

The exclusion criteria include.

- Contraindication to spinal or regional anaesthesia;
- Known hypersensitivity to trial-related medications;
- Significant cardiovascular or pulmonary disease;
- Suffered from severe liver or kidney dysfunction;
- Having a history of significant mental or other health conditions, or an inability to communicate, which precludes assessment or examination;
- Past medical history of neurological deficits;
- Chronic pain conditions or current use or abuse of opioids or alcohol.

### 2.4 Primary outcome

The primary outcome is postoperative pain intensity, assessed using the Visual Analogue Scale (VAS) during standardized movement (instructing patients to turn over) at 12, 24, 48, and 72 h post-surgery. The VAS pain score ranges from 0 (indicating no pain) to 10 (indicating intolerable pain) points. In this longitudinal analysis, a generalized estimating equation (GEE) model with exchangeable correlation structure will be employed to evaluate overall differences in pain scores among groups across the four predefined postoperative intervals.

### 2.5 Secondary outcomes

Secondary outcomes will include patient self-assessment of the Quality of Recovery-15 (QoR-15) scale at 24 and 48 h postoperatively, as well as Visual Analog Scale (VAS) pain scores at rest, recorded at 12, 24, 48, and 72 h post-surgery. Additionally, the number of postoperative acute analgesic requests will be documented. Postoperative anxiety regarding the hypothetical repeat cesarean section will be assessed at 72 h post-surgery using the VAS score (scores range from 0 to 10, with 0 indicating the best and 10 indicating the worst, named VAS anxiety score) (19). Subjective sleep quality will be evaluated for 24, 48, and 72 h post-surgery using the Numerical Rating Scale (NRS) (scores range from 0 to 10, with 0 indicating the worst and 10 indicating the best sleep quality). The maximum walking duration, defined as the longest duration a patient can walk after surgery, will be measured at 24, 48, and 72 h post-surgery. Patient satisfaction with analgesic management will be assessed at 24, 48, and 72 h post-surgery using the NRS (scores range from 0 to 10, with 0 indicating the worst and 10 indicating the best). Postoperative opioid use (including sufentanil via PCIA and any rescue oxycodone) will be recorded over the 0–72 h period. For comparability, all opioid doses will be converted to intravenous morphine milligram equivalents (MME) using standard equianalgesic conversion factors. Both the raw consumption of sufentanil and oxycodone as well as the total MME will be reported. Furthermore, the incidence of nausea and vomiting and length of hospital stay will be recorded.

### 2.6 Safety outcomes

Safety outcomes will assess postoperative adverse reactions including, allergy, wound infection, lightheadedness, headache, circumoral numbness, tongue paresthesia, drowsiness, irritability, hypotension, hypertension, hypoventilation, bradycardia, tachycardia, arrhythmia, and cardiac arrest.

### 2.7 Randomization and blinding

This open-label, three-arm randomized trial allocated 201 participants in a 1:1:1 ratio (LB, R-P, and P groups; *n* = 67 per group). The open-label design was implemented due to fundamental differences in postoperative pain management protocols among the three study groups. Both investigators and participants were unblinded to treatment assignments to facilitate protocol-specified adjustments in postoperative pain management. Postoperative outcome assessments were conducted by trained nurses who were not involved in the intervention process to minimize potential bias.

### 2.8 Anaesthetic and study interventions

All patients fast for at least 8 h and abstain from water for 4 h preoperatively. In the operating room, after establishing a peripheral intravenous channel, they inhale oxygen at 5 L/min via mask, with continuous monitoring of vital signs: non-invasive blood pressure, percutaneous arterial oxygen saturation (SpO_2_), heart rate, and respiratory rate. Preoperatively, all parturients will receive combined spinal-epidural anesthesia. The parturient will be placed in the lateral decubitus position. Following standard aseptic skin preparation and draping with a sterile aperture sheet, subarachnoid blockade will be performed. At the L2/3 or L3/4 interspace, 10–14 mg of 0.5% ropivacaine will be administered intrathecally to achieve rapid onset of surgical anesthesia. An epidural catheter will then be inserted for subsequent intraoperative supplementation if required. In case of inadequate block height or duration, incremental boluses of 2% lidocaine (3-5 mL) or 1% ropivacaine (5 mL) will be administered through the epidural catheter, with careful titration based on the patient’s response and hemodynamic status. Continuous monitoring will be maintained for signs of local anesthetic systemic toxicity, such as tinnitus, perioral numbness, or altered mental status. For parturients with extreme body habitus or height (affecting drug distribution and blockade levels), local anesthetic dosing will be precisely calibrated based on individual anthropometric measurements to optimize analgesic efficacy and safety. Dosing will be precisely calibrated in accordance with each patient’s anthropomorphic measurements to ensure optimal analgesic outcomes. Upon verification of both the requisite analgesic level (at least T6) and the efficacy of the blockade, the cesarean section will be initiated. Supine hypotensive syndrome and total spinal anesthesia are medical emergencies requiring immediate treatment, so, intensive monitoring during the surgical procedure and maintaining hemodynamic stability with the utilization of vasoactive medications as needed to support vital sign parameters are essential. All patients will receive postoperative analgesic regimens as dictated by the sealed-envelope protocol. This information is exclusively known to the anesthesiologist administering the anesthesia, with all other individuals involved remaining blinded to the details. Participants in group LB will receive bilateral QL nerve block [posterior QL block (20)] utilizing liposomal bupivacaine injection under ultrasound guidance (Liposomal bupivacaine injection, 133 mg per 20 mL on each side); participants enrolled in group R-P will receive bilateral QL nerve block utilizing ropivacaine injection under ultrasound guidance (Ropivacaine injection, 20 mL of 0.375% ropivacaine on each side), in conjunction with a PCIA; and participants enrolled in group P will be managed with standard PCIA, without supplementary nerve block interventions. In the preparation of PCIA pumps, each total volume is standardized at 100 mL, and the formulation includes sufentanil and 8 mg of ondansetron. The total amount of sufentanil is calculated based on a dosage of 2 μg per kilogram of body weight. The background infusion rate is set within the range of 0.04 μg/kg/h. The PCIA protocol is set with a 0.04 μg/kg bolus dose and a 15-min lockout interval to ensure safety.

This study will establish an emergency analgesia team to promptly address urgent and intolerable pain, ensuring swift relief for patients. The acute pain management protocol will incorporate paracetamol and oxycodone; initially, intravenous paracetamol 1 g will be administered every 6 h as needed, with a maximum daily dose not exceeding 4 g. If patients continue to report intolerable pain (a VAS pain score > 4) for more than 30 min, or if the maximum daily dose of paracetamol has been reached, oral oxycodone 5 mg will be subsequently employed, with dose adjustments made within the safe dosage range as appropriate. Repeat doses will be given at 4–6 h intervals, with careful monitoring, and dose adjustments within safe limits.

### 2.9 Data collection and monitoring

Data collection will encompass a range of patient characteristics, including age, height, weight, body mass index (BMI), ASA score, drinking status, smoking status, parity, duration of surgery, intraoperative bleeding, and intraoperative analgesia grade. All pertinent data will be meticulously recorded in case report forms (CRFs) and subsequently inputted into an electronic database under the vigilant supervision of the principal investigator (Table 1). An independent Data Monitoring Committee (DMC) will be tasked with the ongoing oversight of the data collection process. Upon completion of data registration, the electronic database will be safeguarded. De-identified datasets will then be forwarded to an independent statistician for comprehensive analysis in accordance with a predefined statistical plan. Any serious adverse events (SAEs), whether or not they are related to the study medication (e.g., persistent hemodynamic instability), must be promptly reported to the principal investigator. In such instances, the perioperative care team is obligated to implement necessary measures to safeguard participant wellbeing. Furthermore, these SAEs must be communicated to the DMC within a 24-h window for thorough discussion and to determine if any adjustments to the study interventions or the study’s discontinuation are warranted.

**Table 1:** Schedule of patient enrolment, study interventions and outcome assessment.

	Study period							
	Enrollment	Allocation	Post-allocation					Close-out
Time point	Pre-op visit	Pre-op 1 day	0 h post-op	12 h post-op	24 h post-op	48 h post-op	72 h post-op	Discharged
Patient enrolment								
Eligibility criteria	×							
Written informed	×							
Consent								
Demographic data	×							
Baseline characteristics	×							
Randomization/allocation		×						
Study interventions								
Liposomal bupivacaine			×					
Ropivacaine & PCIA			×					
PCIA			×					
Outcome assessment								
VAS pain score during turning over				×	×	×	×	
VAS pain score at rest				×	×	×	×	
QoR-15 scale					×	×		
Need for rescue analgesia				×	×	×	×	
VAS anxiety score							×	
NRS sleep score					×	×	×	
Maximum walking duration					×	×	×	
NRS for satisfaction with analgesic					×	×	×	
Opioid consumption								×
Length of hospital stay								×
Nausea			×	×	×	×	×	
Vomiting			×	×	×	×	×	
Adverse events								
Allergy			×	×	×	×	×	
Wound infection			×	×	×	×	×	
Lightheadedness			×	×	×	×	×	
Headache			×	×	×	×	×	
Circumoral numbness			×	×	×	×	×	
Tongue paresthesia			×	×	×	×	×	
Drowsiness			×	×	×	×	×	
Irritability			×	×	×	×	×	
Hypotension			×	×	×	×	×	
Hypertension			×	×	×	×	×	
Hypoventilation			×	×	×	×	×	
Bradycardia			×	×	×	×	×	
Tachycardia			×	×	×	×	×	
Arrhythmia			×	×	×	×	×	
Cardiac arrest			×	×	×	×	×	

According to SPIRIT, statement of defining standard protocol items for clinical trials.

Pre-op, preoperative; Post-op, postoperative; QoR-15, patient self-assessment of Quality of Recovery-15 scale; VAS, visual analogue scale; NRS, numerical rating scale; PCIA, patient-controlled intravenous analgesia pump.

### 2.10 Sample size calculation

Based on previous studies (21–23), a minimum change of at least 1.40 in the VAS pain score is considered to be clinically significant, denoting a substantial alteration in pain perception. The standard deviation (SD) of 1.55, derived from these studies, quantifies the variability in pain measurements. Using a two-sided significance level (*α*) of 0.05 and a desired statistical power of 80%, the initial sample size calculation based on a *t*-test indicated that a minimum of 20 participants per group would be necessary to detect a clinically significant change. This calculation assumed a single measurement per patient. However, the primary analysis includes 4 repeated measurements per patient, which increases the statistical power. To account for the correlation among repeated measures, we incorporated an intraclass correlation coefficient (ICC) of 0.53 into the sample size calculation. Additionally, to address multiple pairwise comparisons among the three study groups, we applied the Bonferroni correction, adjusting the significance level from 0.05 to 0.017 (0.05/3). The final sample size was calculated using the following formula for repeated measures: The formula is $n=\frac{{\left(Z1-\alpha /2+Z1-\beta \right)}^{2}\times (1+(\kappa -1)\rho )\times \sigma^{2}}{\Delta^{2}\times (1-\rho )}$ (*n* is the sample size required per group, κ is the number of repeated measurements (4 in this study), σ is the standard deviation (1.55), Δ is the minimal clinically important difference (1.40), and ρ is the intraclass correlation coefficient (0.53). After adjusting for repeated measures, Bonferroni correction, and an anticipated dropout rate of 10%, we determined that a sample size of 67 participants per group would be required. Therefore, we plan to enroll a total of 201 patients (67 per group).

### 2.11 Statistical analysis

The normality of continuous variables will be assessed using the Shapiro–Wilk test. Data following a normal distribution will be reported as mean (standard deviation, SD), while non-normally distributed data will be presented as median (interquartile range, IQR). Binomial variables will be expressed as proportions. A generalized estimating equation (GEE) model will be used to analyze repeated measures of continuous variables, including VAS scores, QoR-15 scores, maximum walking duration, and subjective sleep quality, accounting for within-subject correlations. The correlation structure will be selected based on the quasi-likelihood under the independence model criterion (QIC). Data distribution will determine the use of either a normal or gamma distribution with identity or log-link functions. *Post hoc* pairwise comparisons will be Bonferroni-corrected. Continuous variables measured at a single time point, such as hospital stay and sufentanil consumption, will be analyzed using *t*-tests or Mann–Whitney U tests depending on normality. Categorical data will be analyzed using the *χ*^2^ test, with *p*-values adjusted using the Bonferroni method and set at 0.017 for pairwise comparisons. Statistical significance will be defined as *p* < 0.05.

All study outcomes will be analysed in the modified intentiontotreat population, including all patients who undergo randomisation with relevant data available. Patients will be included in the analysis according to their original allocation. Multiple testing corrections for secondary outcomes will not be applied; therefore, these outcomes should be interpreted as exploratory findings. Odds ratios (OR) and corresponding 95% confidence intervals (CI) will be reported where appropriate. No interim analysis will be planned. Missing data will not be imputed. Statistical analyses will be performed using SPSS (version 25.0; IBM SPSS). A two-sided *p*-value < 0.05 will indicate statistical significance, except where false discovery rate (FDR) corrections apply.

### 2.12 Patient and public involvement

Patients and the public will not participate in the study’s design, recruitment, conduct, or reporting. Study results will be shared with participants via email.

## 3 Discussion

In this prospective, randomized, open-label, controlled clinical trial, we will recruit 201 adult parturients undergoing cesarean delivery to assess the efficacy and safety of bilateral QL block with liposomal bupivacaine for post-cesarean analgesia. This study will be implemented in accordance with the Consolidated Standards of Reporting Trials guidelines and address three critical clinical questions regarding the proposed postoperative analgesic strategy. First, whether it fulfills fundamental analgesic requirements. Second, whether it independently achieves contemporary standards for comprehensive pain management. And, whether it confers clinically significant co-benefits beyond analgesia. The primary outcome is postoperative pain intensity, evaluated using the VAS pain score during standardized movement at 12, 24, 48, and 72 h post-surgery. Secondary outcomes include quality of recovery (QoR-15 scores), VAS scores at rest, need for rescue analgesia, postoperative anxiety regarding a hypothetical repeat cesarean section, subjective sleep quality, maximum walking duration, patient satisfaction with analgesia, postoperative nausea and vomiting, opioid consumption, and hospital length of stay.

The three-arm design of this trial was structured to address two key clinical questions regarding post-cesarean analgesia. First, by comparing bilateral QL block with liposomal bupivacaine to standard intravenous analgesia alone, the study aims to evaluate whether a single-injection, long-acting regional technique can provide superior pain control and reduce reliance on systemic opioids. Second, the inclusion of a group receiving QL block with ropivacaine combined with intravenous analgesia allows for a direct comparison between liposomal and conventional local anesthetics in terms of both analgesic efficacy and opioid-sparing potential. This design facilitates a comprehensive assessment of the relative benefits of QL block strategies, not only in improving postoperative pain outcomes but also in minimizing opioid-related adverse effects.

Liposomal bupivacaine, an extended-release formulation, prolongs local anesthetic effects for at least 72 h by controlling the release of encapsulated bupivacaine (15). Its clinical efficacy and safety have been well-documented in multiple surgical settings, demonstrating superior pain control and opioid-sparing effects (24, 25). A study on post-hemorrhoidectomy pain found that liposomal bupivacaine, when administered via local infiltration anesthesia, extended analgesia to an average of 14.3 h before additional analgesia was required, compared to 1.2 h in the control group (26). When administered via TAP blocks, this formulation achieved a maximum complete analgesia duration of 15 h, significantly exceeding conventional local anesthetics, furthermore, those patients exhibited superior recovery metrics during the 72-h postoperative period (27). Notably, its administration via bilateral TAP nerve blocks has significantly reduced opioid consumption following cesarean delivery (28). However, relevant clinical data on the application of liposomal bupivacaine in bilateral QL blocks are lacking. A critical safety consideration is the potential transfer of bupivacaine into breast milk; studies indicate that the relative neonatal dosage remains below 1%, well within safe limits (14). These findings reinforce the potential of liposomal bupivacaine as an effective and safe component of postoperative pain management, particularly in opioid-reduction strategies.

Advancements in regional anesthesia techniques continue to refine multimodal analgesia strategies. Evidence suggests that QL blocks provide superior and prolonged analgesia compared to TAP blocks in lower abdominal surgeries, including cesarean sections (18). Cadaveric studies demonstrate extensive dye dispersion beyond the transverse processes and nerve roots, further supporting the hypothesis that QL blocks may provide broader and longer-lasting analgesic effects (20). MRI studies in volunteers have also shown that contrast injected via the QL block approach achieves extensive paravertebral spread, reinforcing its potential superiority over TAP blocks (29). Given these findings, the bilateral QL block was selected for this study to optimize postoperative pain control. This study aims to evaluate whether bilateral QL blocks with liposomal bupivacaine can optimize postoperative pain management while reducing analgesic consumption and adverse events in cesarean delivery patients. Methodologically, the investigation will first compare this intervention with standard PCIA to determine if it offers additional benefits in postoperative pain control and reduces the incidence of adverse reactions. Subsequently, the liposomal bupivacaine-based bilateral QL block strategy will be further contrasted with the R-P group (a multimodal analgesia regimen combining regional techniques and pharmacologic agents) to assess whether it achieves comparable clinical benefits to established multimodal protocols. We hypothesize that bilateral QL nerve blocks with liposomal bupivacaine will provide superior postoperative analgesia, reduce opioid consumption, and offer improved safety and practicality. If validated, this approach could significantly optimize post-cesarean recovery protocols and mitigate analgesic-related adverse effects.

Beyond its clinical impact, this study also aims to explore the potential societal implications of effective postoperative pain management. By mitigating postpartum pain and anxiety, improved analgesic strategies may positively influence fertility intentions and maternal wellbeing (3). Addressing postpartum pain through optimized regional anesthesia techniques may help alleviate childbirth-related fears, contributing to a more positive childbirth experience and possibly influencing broader public health trends related to reproductive choices (30).

### 3.1 Limitations

This study has several limitations. First, it is a single-center study with a relatively small sample size, which may limit the generalizability of the findings. Larger, multicenter studies are needed to validate the results. Second, the follow-up period is restricted to 72 h postoperatively, potentially missing long-term analgesic effects and delayed complications. Future studies with extended follow-up are necessary to better assess the prolonged impact of liposomal bupivacaine in cesarean analgesia. Third, this study excludes patients with chronic opioid use or significant comorbidities, which may further restrict its applicability to a broader patient population. Expanding inclusion criteria in future research will be essential to enhance external validity and clinical relevance. Fourth, due to the specific research objectives and the nature of the intervention, an open-label design is adopted instead of blinding. This may introduce risks of several biases, including assay bias, detection bias, and reporting bias, particularly for subjective outcomes. Finally, we did not account for the potential influence of preoperative anxiety, depression, or other psychiatric comorbidities that could affect postoperative pain perception. Future studies should consider evaluating the impact of these psychological factors on analgesic outcomes to provide a more comprehensive assessment of pain management strategies.

## 4 Conclusion

This randomized controlled trial aims to provide high-quality evidence on the efficacy and safety of liposomal bupivacaine QL blocks for post-cesarean analgesia, with a specific focus on opioid reduction and enhanced recovery. The findings are expected to support the wider clinical adoption of this approach, potentially improving postoperative outcomes. Furthermore, this study highlights the broader implications of pain management in the context of maternal health and reproductive choices, emphasizing the role of effective analgesic strategies in mitigating childbirth-related anxiety. Future research should continue to explore the long-term benefits and wider applicability of this approach across diverse surgical populations.
